# Patterned Clues in an Infant With Annular Rash and Seizures Revealing Kawasaki Disease

**DOI:** 10.1155/crpe/8346863

**Published:** 2026-06-25

**Authors:** Islam Elmitwalli, John A. Ruth, Gabriela Moraru

**Affiliations:** ^1^ Department of Pediatrics, Oklahoma Children’s Hospital, Oklahoma City, Oklahoma, USA; ^2^ Department of Pediatrics, Division of Infectious Disease, Oklahoma Children’s Hospital, Oklahoma City, Oklahoma, USA

**Keywords:** annular rash, aseptic meningitis, infant rash, Kawasaki disease

## Abstract

Kawasaki disease (KD) is a systemic vasculitis primarily affecting children, but its presentation can be highly variable, especially in young infants. While KD is classically associated with a polymorphous rash, annular or targetoid rashes are rare and can contribute to diagnostic uncertainty. Additionally, neurological complications such as seizures and aseptic meningitis are uncommon, further complicating recognition. We present a case with these atypical features, which closely mimic other conditions, including meningoencephalitis and reactive infection mucocutaneous eruption in children.

## 1. Introduction

Kawasaki disease (KD) is an acute febrile vasculitis that is relatively rare in infants under 6 months of age. The peak incidence of KD occurs between 6 months and 2 years old, with only about 3%–10% of cases occurring in infants under 6 months old [1]. These infants often present with more incomplete features, with varying reports showing that 35%–88% of infants less than 6 months of age present with incomplete KD [2, 3]. Incomplete KD refers to cases where the patient does not meet the full diagnostic criteria for classic KD, which includes a fever lasting ≥ 5 days and at least four of the following five features: conjunctivitis, oral mucosal changes, polymorphous rash, extremity changes, and cervical lymphadenopathy [1]. KD in early infancy can present with diagnostic difficulties that arises from the atypical and less common presentations, as well as the overlap with other infections and diseases that share nonspecific symptoms. Here, we present a rare case of incomplete KD in an infant who presented with atypical features of convulsions and rash resembling erythema multiforme.

## 2. Case Description

**FIGURE 1:**
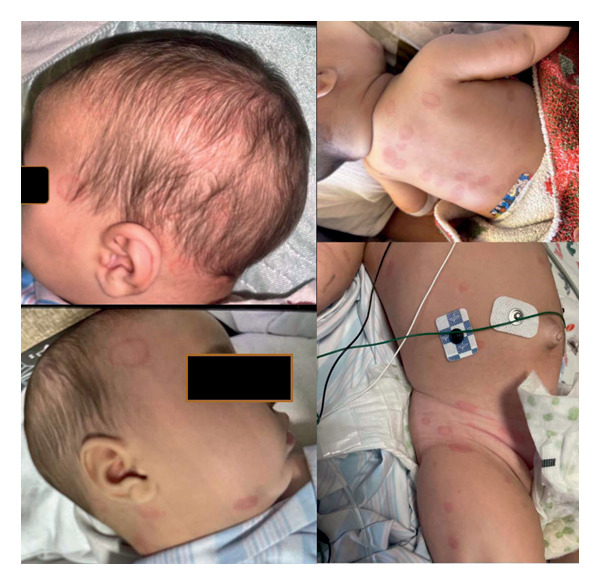
Diffusely distributed target‐like lesions over her head, face, torso, and extremities.

A 5‐month‐old previously healthy female presented with a high‐grade fever for five days (102–106°F) and a widespread annular/targetoid rash, cracked lips, and nonpurulent conjunctivitis. In the 24 h preceding her evaluation, she developed two episodes of full body stiffening lasting 45 s, accompanied by horizontal nystagmus and postictal drowsiness, prompting the visit to the emergency department. The patient had been diagnosed with SARS‐CoV‐2 the day before presentation to our hospital, though no cough or congestion was reported. On physical examination, she was extremely irritable but alert and responsive, with intact cranial nerves, appropriate motor function, and no focal deficits. Skin evaluation showed diffusely distributed target‐like lesions over her head, face, torso, and extremities, with discrete nonexudative conjunctivitis and erythematous lips. The skin findings are shown in Figure 1. No neck rigidity, extremity edema, or cervical lymphadenopathy was noted. The rest of the exam was unremarkable. Our patient lacked prior SARS‐CoV‐2 immunization and has no in utero exposure to SARS‐CoV‐2 vaccination.

Laboratory findings revealed a white blood cell count of 20.8 × 10^3^/μL with an absolute neutrophil count of 13.7 × 10^3^/μL. Inflammatory markers were elevated, with C‐reactive protein (CRP) at 151 mg/L and procalcitonin at 5.6 ng/mL, indicating systemic inflammation. Albumin levels and renal and hepatic function tests were within normal limits, ruling out significant organ dysfunction. Pro–B‐type natriuretic peptide was markedly elevated with 15,323 pg/mL. Cerebrospinal fluid (CSF) analysis showed mild pleocytosis (WBC: 245 cells/μL; RBC: 15 cells/μL), with 77% neutrophils, proteinorachia of 108 mg/dL, and normal glucose of 69 mg/dL, suggestive of aseptic meningitis. Blood culture, CSF culture, and CSF PCR for enterovirus, EBV, and HSV were negative, as well as CSF multiplex PCR including *E. coli* K1, *H. influenzae*, *L. monocytogenes*, *N. meningitidis*, *S. agalactiae*, *S. pneumoniae*, *Cytomegalovirus* (CMV), enterovirus, human herpes HSV‐1/2 and HHV‐6, *Parechovirus*, varicella‐zoster virus (VZV), and *Cryptococcus* spp. Nasopharyngeal swab and serology were negative for *Mycoplasma pneumoniae*. However, empiric antibiotics (ceftriaxone, vancomycin, and acyclovir) were given for suspected sepsis/meningitis prior to collection of the CSF and blood cultures. Nasopharyngeal PCR testing for rhinovirus/enterovirus was positive, though SARS‐CoV‐2 was negative. Brain MRI was normal, helping to rule out an acute intracranial event. Chest X‐ray demonstrated perihilar opacities and streaky opacities in the medial lower lungs, raising concern for developing pneumonia/atelectasis. Given the negative MRI, atypical KD was considered, and treatment with high‐dose intravenous immunoglobulin (IVIG) and aspirin was initiated by Day 7 of illness. An echocardiogram was ordered and revealed normal cardiac function with a borderline dilation of the right coronary artery (*Z*‐score: 1.94, below the threshold of 2). Subsequently, the patient showed significant improvement, with resolution of fever and improvement in inflammatory markers. She did not require additional therapy.

## 3. Discussion

Our patient was diagnosed with incomplete KD with atypical features of aseptic meningitis, a rare neurological manifestation of KD. Neurological complications in KD have been reported in approximately 1%–5% of cases and can include irritability, lethargy, headaches, seizures, aseptic meningitis, encephalopathy, and, in rare cases, stroke [4–6]. Seizures are particularly uncommon, with an incidence ranging from 0.18% to 0.46%, and tend to occur more frequently in infants younger than 6 months [6, 7]. Although KD involves a robust proinflammatory cytokine response, the pathophysiology of seizures in this context appears to differ from typical febrile seizures [8]. Available literature suggests that seizures in KD are more often related to aseptic meningitis or encephalitis rather than to cytokine‐triggered febrile responses [8]. Moreover, febrile seizures appear to be even less common in KD than in the general pediatric population [7]. Cases with KD and aseptic meningitis usually have an overlapping symptomatology with bacterial meningitis, including fever, irritability, elevated inflammatory markers (CRP and ESR), and CSF pleocytosis. It is easy to misdiagnose KD as bacterial meningitis, particularly in the early infancy. A key distinguishing factor is that CSF in KD patients is sterile (culture‐negative), demonstrates pleocytosis, has normal glucose levels, and may show mild protein elevation [6]. In our patient, the presence of elevated inflammatory markers, CSF pleocytosis with normal glucose, and the absence of an identifiable common bacterial pathogens helped rule out bacterial meningitis and supported the diagnosis of atypical KD with aseptic meningitis. Atypical KD is distinct from the term incomplete KD. Incomplete KD refers to patients who do not fulfill all classic diagnostic criteria for KD. In contrast, atypical KD describes patients who meet the classic criteria for KD but present with additional uncommon manifestations, as renal impairment, hepatitis, pancreatitis, testicular swelling, or meningitis [9].

In KD, a key diagnostic criterion is the presence of a polymorphous rash, which is typically a diffuse, nonvesicular eruption that may present as maculopapular or urticarial. Although rare in KD, an annular rash has been described in a few reports [10–14]. Some of these reports have suggested that *Streptococcus pyogenes* may play a role in the pathogenesis of KD and contribute to the development of the annular rash, possibly through a superantigen‐mediated mechanism [11, 12]. Concurrent infections are frequently observed in KD. Various bacterial and viral pathogens have been reported, causing soft tissue infections, pneumonia, joint infections, and other manifestations alongside KD [15–18]. The incidence of such coinfections can be as high as 10%–16% [15, 19]. The presence of accompanying infections should not deter clinicians from considering KD when the clinical presentation is consistent with the diagnosis. Concurrent infection with SARS‐CoV‐2 has been described to play a role in the pathogenesis of the systemic inflammatory response observed in KD, as well as in patients presenting with atypical features, including seizures at a young age [20–22]. Acute SARS‐CoV‐2 infection may trigger hyperactivation of Fc receptors on mast cells, leading to an aberrant immune response, especially in patients with prior COVID‐19 vaccination [13, 23, 24]. Although our patient was diagnosed with SARS‐CoV‐2 infection one day before presenting to our hospital, we believe it is less likely that SARS‐CoV‐2 contributed to the development of KD. Our patient lacked clinical features suggestive of acute SARS‐CoV‐2 infection and had a negative PCR test at our facility.

The annular pattern of skin rash that resembles erythema multiforme is more commonly associated with infections such as *Mycoplasma pneumoniae*. *Mycoplasma pneumoniae* infections have been associated with rashes and mucocutaneous manifestations such as urticaria, erythema multiforme, Stevens–Johnson syndrome (SJS), and *Mycoplasma pneumoniae*–induced rash and mucositis (MIRM) syndrome [25]. In MIRM, the mucocutaneous eruptions often involve both the skin and mucous membranes, including oral, ocular, and genital sites. The skin findings typically present as annular targetoid patterns or vesiculobullous lesions [25]. The diagnostic evaluation did not support the possibility of MIRM or *Mycoplasma pneumoniae* infection, as both the nasal swab and serum antibody IGM and IGG testing for *Mycoplasma* were negative. Pulmonary involvement is not a common feature of KD or incomplete KD, with pulmonary changes documented in less than 2% of the patients [26]. These children initially present with fever, cough, and respiratory distress, with imaging findings suggestive of pneumonia‐like changes. Suspicion for KD is often raised when fever persists despite antibiotic treatment, especially in the settings of thrombocytosis and elevated ESR and CRP levels. Other pulmonary findings may include nodules, bronchitis, pleural effusion, and pneumothorax [26]. Children with KD and pneumonia‐like changes had a higher risk of developing coronary artery lesions compared to those without pulmonary involvement. In a retrospective randomized study including 976 patients with KD, Liu et al. investigated the rate of cardiac complications in KD and found that patients with pneumonia‐like radiographic findings had nearly twice the risk of developing coronary artery lesions compared to those without radiologic changes [27].

Our case of atypical KD in a 5‐month‐old infant was particularly challenging to diagnose due to overlapping features with bacterial meningitis, encephalitis, pneumonia, MIRM, and *Mycoplasma pneumoniae* infection. Younger infants with KD are at higher risk for serious cardiac complications than other age group, including coronary artery aneurysms, coronary artery dilation, and myocardial dysfunction. These potential complications should be considered as early as possible to initiate therapy with high‐dose IVIG and aspirin to reverse the systemic inflammation and prevent irreversible cardiac injury.

## Funding

This research did not receive any specific grant from agencies in the public, commercial, or not‐for‐profit sectors.

## Disclosure

This work was previously presented as an abstract titled “A rare presentation of Kawasaki disease: annular rash and aseptic meningitis in an infant,” published in February 2026 in Southern Regional Meeting [28]. The current manuscript includes additional clinical details, expanded discussion, and updated literature review not included in the abstract.

## Ethics Statement

This case report did not require Institutional Review Board approval in accordance with institutional policies. Written informed consent was obtained from the patient’s legal guardian for publication of this case report and accompanying clinical information.

## Conflicts of Interest

The authors declare no conflicts of interest.

## Data Availability

Data sharing is not applicable to this article as no datasets were generated or analyzed during the current study.
